# Risk factors for major complications following colorectal resections for endometriosis in the USA

**DOI:** 10.1007/s00384-023-04577-5

**Published:** 2023-12-06

**Authors:** Raanan Meyer, Yosef Y. Nasseri, Moshe Barnajian, Matthew T. Siedhoff, Kelly N. Wright, Kacey M. Hamilton, Gabriel Levin, Mireille D. Truong

**Affiliations:** 1 Division of Minimally Invasive Gynecologic Surgery, Department of Obstetrics and Gynecology, Cedars-Sinai Medical Center, Los Angeles, CA USA; 2 The Dr. Pinchas Bornstein Talpiot Medical Leadership Program, Sheba Medical Center, Tel HaShomer, Ramat-Gan, Israel; 3 Department of General Surgery, Cedars-Sinai Medical Center, Los Angeles, CA USA; 4 Lady Davis Institute for Cancer Research, Jewish General Hospital, McGill University, Quebec, Canada

**Keywords:** Laparoscopy, Laparotomy, Minimally invasive surgery, Postoperative complications

## Abstract

**Purpose:**

We aimed to describe the incidence and identify risk factors for the occurrence of short-term major posto-perative complications following colorectal resection for endometriosis.

**Methods:**

A cohort study using data from the American College of Surgeons National Surgical Quality Improvement Program (NSQIP) database from 2012–2020. We included patients with a primary diagnosis of endometriosis who underwent colon or rectal resections for endometriosis.

**Results:**

Of 755 women who underwent colorectal resection, 495 (65.6%) had laparoscopic surgery and 260 (34.4%) had open surgery. The major complication rate was 13.5% (*n* = 102). Women who underwent open surgery had a higher proportion of major complications (*n* = 53, 20.4% vs. *n* = 49, 9.9%, *p* < 0.001). In a multivariable regression analysis, Black race (aOR 95%CI 2.81 (1.60–4.92), *p* < 0.001), Hispanic ethnicity (aOR 95%CI 3.02 (1.42–6.43), *p* = 0.004), hypertension (aOR 95%CI 1.89 (1.08–3.30), *p* = 0.025), laparotomy (aOR 95%CI 1.64 (1.03–3.30), *p* = 0.025), concomitant enterotomy (aOR 95%CI 3.02 (1.26–7.21), *p* = 0.013), and hysterectomy (aOR 95%CI 2.59 (1.62–4.15), *p* < 0.001) were independently associated with major post-operative complications. In a subanalysis of laparoscopies only, Hispanic ethnicity, chronic hypertension, lysis of bowel adhesions, and hysterectomy were independently associated with major complications. In a subanalysis of laparotomies only, Black race and hysterectomy were independently positively associated with the occurrence of major complications.

**Conclusion:**

This study provides a current population-based estimate of short-term complications after surgery for colorectal endometriosis in the USA. The identified risk factors for complications can assist during preoperative shared decision-making and informed consent process.

**Supplementary Information:**

The online version contains supplementary material available at 10.1007/s00384-023-04577-5.

## Introduction

Endometriosis is a chronic disease characterized by the presence of endometrial glands and stroma outside of the uterus [1]. It affects 7–10% of the female population, most commonly of reproductive age. The most common clinical presentations are dysmenorrhea, infertility, and adnexal masses. Sites of endometriosis dissemination include the ovaries, anterior and posterior cul-de-sac, broad ligament, and uterosacral ligaments [2, 3]. It is estimated that between 5 and 25% of women with endometriosis have colorectal endometriosis, although this estimation is limited by referral bias [4–6]. Common symptoms of colorectal endometriosis include dyschezia, rectal bleeding, constipation, bloating, and diarrhea [7–9]. Surgical management of colorectal endometriosis is generally considered in cases of severe symptoms and/or failed medical therapy [10–12]. Surgical options include rectal shaving, disc excision, and segmental resection. Colorectal resection may be the most suitable technique in cases of large bowel infiltration [6]. However, it is also associated with the highest rate of post-operative complications [6, 7]. These include anastomotic leakage, infections, bowel perforation requiring reoperation, and hemorrhage. Studies evaluating outcomes following colorectal resection for endometriosis are mostly of a single-center origin and of limited sample size [6, 12, 13]. Furthermore, data focusing on laparoscopic and open approaches separately are scant, although most endometriosis surgeries were performed laparoscopically in the last two decades [6, 13].

We aimed to use a national database to describe the incidence of short-term major post-operative complications among patients undergoing colectomy or rectal resection for endometriosis and to identify risk factors for complications occurrence.

## Materials and methods

In this retrospective study, we used the data from the American College of Surgeons National Surgical Quality Improvement Program (NSQIP) database. We identified women who underwent surgery for endometriosis between the years 2012 and 2020, identified by the International Classification of Diseases Revision Ninth/Tenth (ICD-9/10) post-procedure codes. The ICD codes included the following: 617.1/N80.1—endometriosis of the ovary, 617.2/N80.2—endometriosis of the fallopian tube, 617.3/N80.3—endometriosis of the pelvic peritoneum, 617.4/N80.4—endometriosis of the rectovaginal septum and vagina, 617.5/N80.5—endometriosis of the intestine, 617.6/N80.6—endometriosis in the scar of the skin/cutaneous scar, 617.8/N80.8—endometriosis of other specified sites/other endometriosis, and 617.9/N80.9—endometriosis, unspecified. Of these cases, we identified cases with the following current procedural terminology (CPT) codes, indicating colon or rectal resection: 44140–44160, 44202–44213, 45110–45123, and 45395–45397. We excluded cases with the ICD 9/10 codes 617.0/N80.0 signifying endometriosis of the uterus, as these cases were thought to represent adenomyosis. Other exclusion criteria included non-elective surgeries, malignancy, vaginal hysterectomies, and women with preoperative sepsis.

We collected baseline and preoperative characteristics, intraoperative characteristics, and post-operative complications. Baseline characteristics and preoperative characteristics included age, race, body mass index (BMI), tobacco use, diabetes mellitus, hypertension requiring medication, chronic obstructive pulmonary disease, immunosuppressive therapy, bleeding disorders, preoperative blood transfusion within 72 h of surgery start time, and American Society of Anesthesiologists (ASA) physical status classification system class. Intraoperative characteristics included the surgical approach, laparoscopy or laparotomy, the procedure performed—colectomy or rectal resection—concomitant procedures performed, and total operative time. Laparoscopic approach included both conventional laparoscopy and robot-assisted laparoscopy, as the latter approach is not reported separately in the NSQIP database. Cases where both laparoscopy and laparotomy approaches were listed based on the CPT codes were defined as laparotomy cases, as the reason for listing both approaches could not be ascertained—e.g., intraoperative complication and recording error.

We categorized any concomitant procedures performed during the surgery into the following: intestinal or rectal procedures (Supplemental Table 1), hysterectomy, myomectomy, ovarian cystectomy, salpingectomy with or without oophorectomy, excision or fulguration of pelvic lesions, and ureterolysis. Definitions were based on CPT codes.

Post-operative complications were classified as minor or major. Major complications included any of the following, occurring within 30 days of surgery: unplanned reoperation, unplanned intubation, mechanical ventilation > 48 h, deep incisional surgical site infection, organ space surgical site infection, wound disruption, blood transfusion (within 72 h of surgery start time), sepsis, septic shock, cerebrovascular accident, pneumonia, deep venous thromboembolism, pulmonary embolism, myocardial infarction, cardiac arrest, renal insufficiency, and length of hospitalization > 30 days or death. Minor post-operative complications included the occurrence of urinary tract infection, superficial incisional surgical site infection, and unplanned readmission.

### Statistics analysis

We performed a descriptive analysis using a chi-squared test and Fisher’s exact test as appropriate. Mann–Whitney *U* test was used to analyze continuous variables. Categorical variables are reported as median with interquartile range and continuous variables as proportions. Multivariable regression analysis was conducted to identify independent parameters associated with the occurrence of major post-operative complications. The regression analysis model included factors that were found to be statistically significant in the univariate analysis and are clinically relevant. The results are reported as adjusted odds ratio (aOR) and 95% confidence interval (CI). We performed sub-analyses for laparoscopy and laparotomy cases. A 2-sided *p*-value < 0.05 was considered statistically significant. Statistical analyses were performed using Software Package for Statistics and Simulation (IBM SPSS version 27, IBM Corp, Armonk, NY).

### Ethical approval

As the data used for this study are publicly available and do not include protected health information, the Institutional Review Board concluded that approval is not required.

## Results

**Fig. 1:**
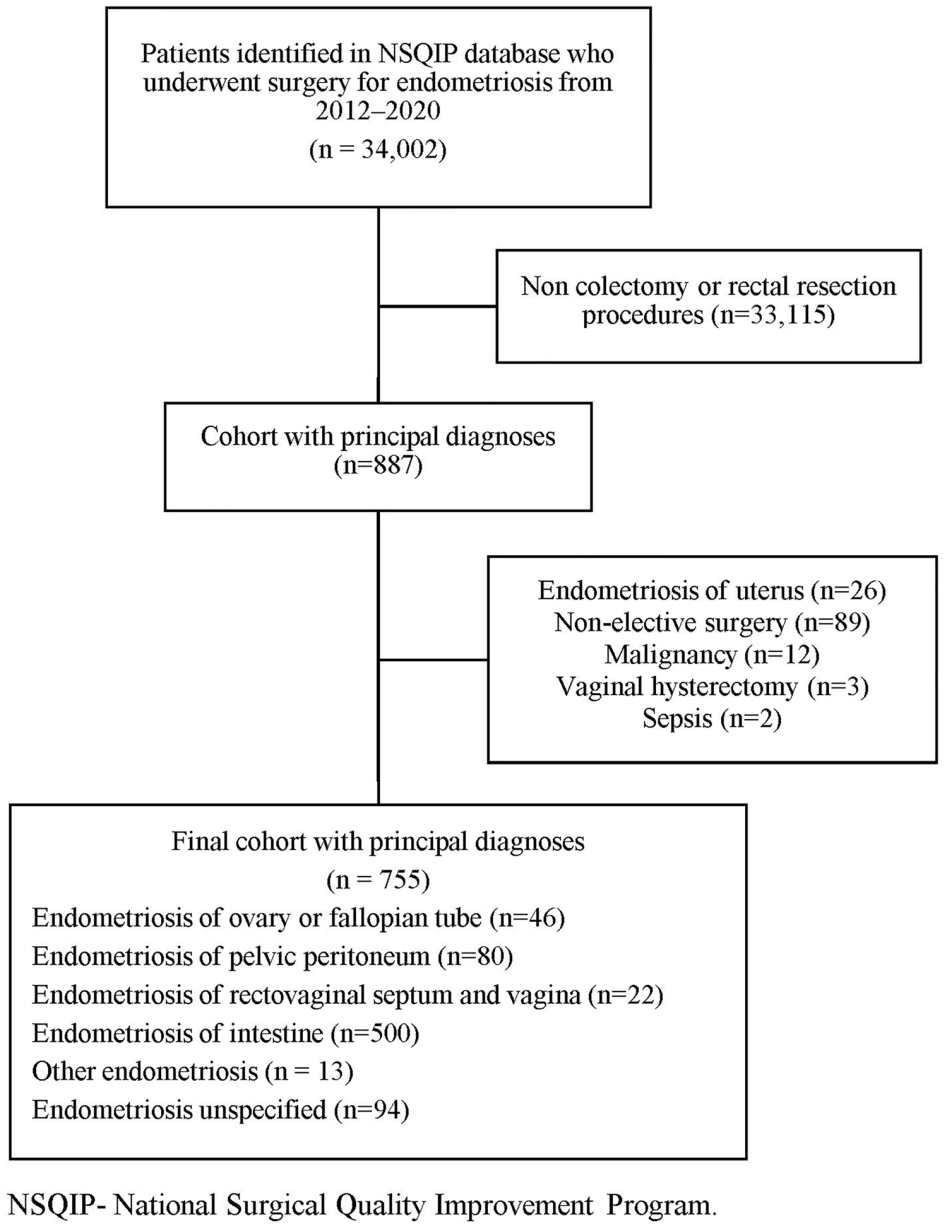
Study population

**Fig. 2:**
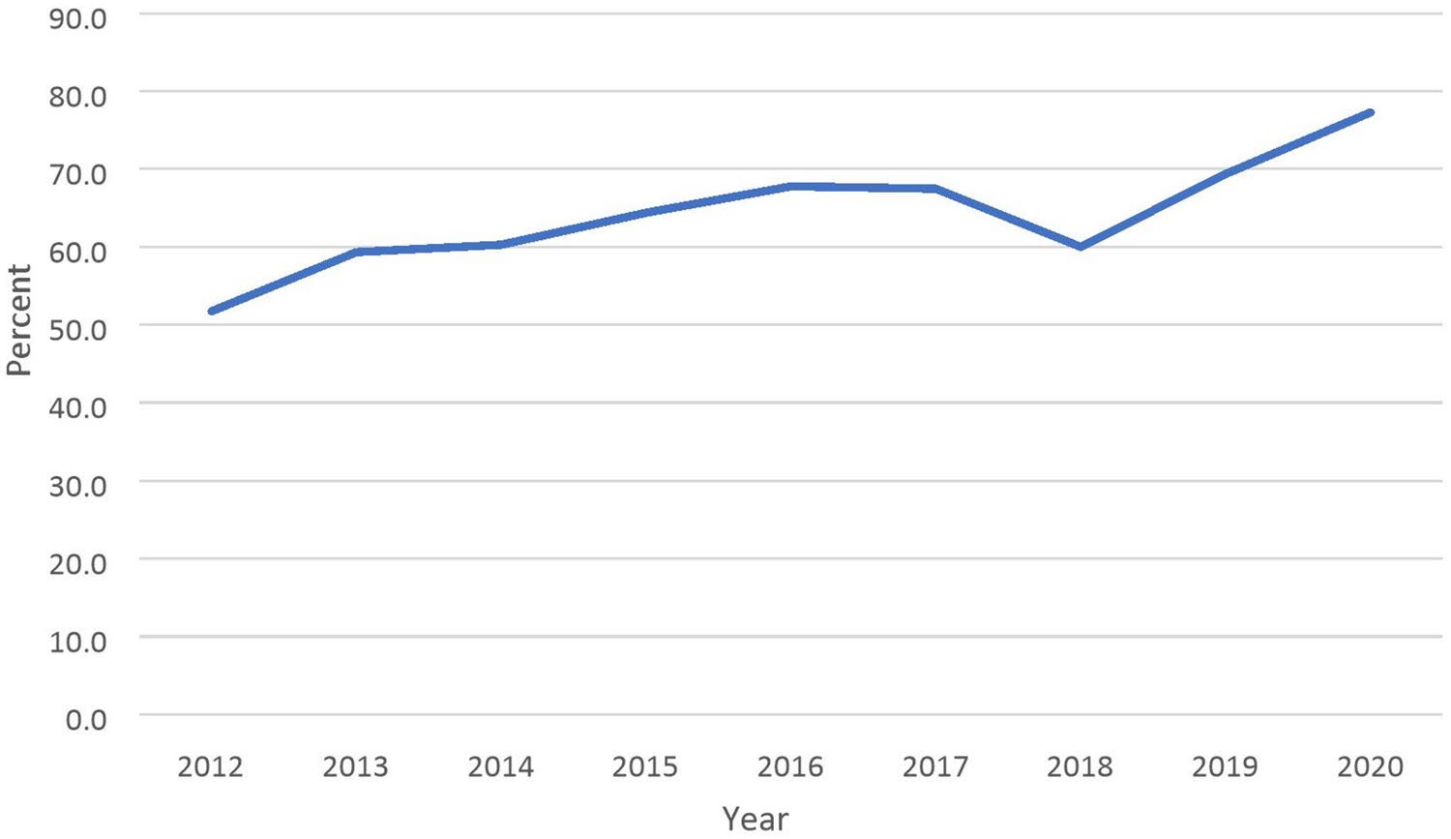
Percentage of laparoscopies of all surgeries performed for colorectal endometriosis

We identified 34,002 women who underwent surgery between 2012 and 2020 in the NSQIP database (Fig. 1). Of those, a total of 755 (2.2%) women underwent colorectal resection for endometriosis and constituted the study’s cohort. Major post-operative complications occurred in 102 (13.5%) women. Laparoscopic surgery was performed in 495 (65.6%) of women, and 260 (34.4%) women underwent laparotomy. The proportion of laparoscopic surgery increased from 51.7% in 2012 to 77.3% in 2020 (Fig. 2). The rate of major complications was lower in laparoscopic cases when compared to laparotomy (*n* = 49/495, 9.9% vs. *n* = 53/260, 20.4% respectively, *p* < 0.001).

Table 1 presents a comparison of women with major complications vs. women without major complications. White race representation was higher in women without major complications (64.2% vs. 39.2%, *p* < 0.001), while Black race representation was higher in the major complications group (30.4% vs. 13.5%, *p* < 0.001). The proportion of hypertension was lower among women without complications (13.8% vs. 23.5%, *p* = 0.011), as well as the proportion of laparotomy (31.7% vs. 52.0%, *p* < 0.001). The proportions of concomitant small bowel enterotomy and lysis of bowel adhesions were lower in the group without major complications (2.9% vs. 11.8%, *p* < 0.001, 13.2% vs. 25.5%, *p* = 0.002, respectively). The proportion of hysterectomy was lower in the group without complications (21.9% vs. 46.1%, *p* < 0.001). Total operative time was shorter in the group without complications (median 204.0 vs. 286.5 min, *p* < 0.001).

**Table 1:** Comparison of women with and without major complications following colorectal resection for endometriosis

**Characteristics**	**No major complications (*n*** **= 653)**	**Major complications (*n*** **= 102)**	***p*-value**
Age, years	40 [35.0–45.0]	40.5 [36.0–44.0]	0.691
Race			
White	419 (64.2)	40 (39.2)	< 0.001
Black or African American	88 (13.5)	31 (30.4)	< 0.001
Hispanic	41 (6.3)	12 (11.8)	0.058
Asian	24 (3.7)	6 (5.9)	0.289
Native Hawaiian or Pacific Islander	3 (0.5)	1 (1.0)	0.441
American Indian or Alaska Native	3 (0.5)	0 (0)	> 0.999
Unknown	75 (11.7)	12 (11.8)	
Body mass index, mean (kg/m^2^)	26.4 [22.8–31.5]	26.4 [23.0–33.2]	0.622
Tobacco use	56 (8.6)	12 (11.8)	0.295
Diabetes mellitus	12 (1.8)	5 (4.9)	0.052
Hypertension	90 (13.8)	24 (23.5)	0.011
Chronic obstructive pulmonary disease	2 (0.3)	1 (1.0)	0.353
Immunosuppressive therapy	13 (4.0)	4 (3.9)	0.269
Bleeding disorders	5 (0.8)	0 (0)	> 0.999
Preoperative transfusion	1 (0.2)	2 (2.0)	0.049
ASA classification			0.644
I	89 (13.6)	11 (10.8)	
II	457 (70.0)	69 (67.6)	
III	105 (16.1)	22 (21.6)	
IV	1 (0.2)	0 (0)	
Colectomy	615 (94.2)	92 (90.2)	0.125
Rectal resection	38 (5.8)	10 (9.8)	
Laparotomy	207 (31.7)	53 (52.0)	< 0.001
Laparoscopy	446 (68.3)	49 (48.0)	
Colostomy	6 (0.9)	2 (2.9)	0.109
Concomitant procedures			
Small bowel enterotomy	19 (2.9)	12 (11.8)	< 0.001
Lysis of bowel adhesions	86 (13.2)	26 (25.5)	0.002
Other bowel procedures	13 (2.0)	4 (3.9)	0.269
Hysterectomy	143 (21.9)	47 (46.1)	< 0.001
Myomectomy	17 (2.6)	5 (4.9)	0.199
Ovarian cystectomy	19 (2.9)	4 (3.9)	0.537
Salpingectomy w/wo oophorectomy	183 (28.0)	56 (54.9)	< 0.001
Excision or fulguration of pelvic lesions	137 (21.0)	23 (22.5)	0.718
Ureterolysis	30 (4.6)	11 (10.8)	0.01
Total operative time (minutes)	204.0 [134.0–294.5]	286.5 [222.8–393.0]	< 0.001

Data are *n* (%) or median [interquartile range]

Blood transfusion was the most common major complication, occurring in 57 (55.9%) of 102 cases with complications (Table 2), followed by unplanned reoperation and organ space surgical site infection (29/102, 28.4% for both) and sepsis (15/102, 14,7%).

**Table 2:** Types of post-operative complications among women with and without major complications following colorectal resection for endometriosis

	**No major complications (*n* = 653)**	**Major complications (*n* = 102)**
Major complications		
Unplanned reoperation		29 (28.4)
Unplanned intubation		1 (1.0)
Ventilation > 48 h		1 (1.0)
Deep incisional surgical site infection		3 (2.9)
Organ space surgical site infection		29 (28.4)
Wound disruption		5 (4.9)
Blood transfusion		57 (55.9)
Sepsis		15 (14.7)
Septic shock		3 (2.9)
Cerebrovascular accident		0 (0)
Pneumonia		4 (3.9)
Deep venous thromboembolism		6 (5.9)
Pulmonary embolism		4 (3.9)
Myocardial infarction		0 (0)
Cardiac arrest		1 (1.0)
Renal insufficiency		3 (2.9)
Length of hospitalization > 30 days		2 (2.0)
Mortality		0 (0)
Minor complications		
Urinary tract infection	10 (1.5)	8 (7.8)
Superficial incisional surgical site infection	19 (2.9)	5 (4.9)
Unplanned readmission related to the operation	25 (3.8)	36 (35.3)
Length of hospitalization (days)	3.0 [3.0–5.0]	5.0 [4.0–8.0]

Data are *n* (%) or median [interquartile range]

In a multivariable regression analysis (Table 3), the following factors were independently associated with major post-operative complications: Black race (aOR 95%CI 2.81 (1.60–4.92), *p* < 0.001), Hispanic ethnicity (aOR 95%CI 3.02 (1.42–6.43), *p* = 0.004), hypertension (aOR 95%CI 1.89 (1.08–3.30), *p* = 0.025), laparotomy (aOR 95%CI 1.64 (1.03–3.30), *p* = 0.025), concomitant enterotomy (aOR 95%CI 3.02 (1.26–7.21), *p* = 0.013), and hysterectomy (aOR 95%CI 2.59 (1.62–4.15), *p* < 0.001) (Table 4).

**Table 3:** Multivariable regression analysis of factors associated with major complications following colorectal resection for endometriosis

	**Odds ratio 95% confidence interval**	***p*-value**
Race and ethnicity		
White	Reference	–
Black or African American	2.81 (1.60–4.92)	< 0.001
Hispanic	3.02 (1.42–6.43)	0.004
Asian	2.19 (0.80–5.95)	0.126
Hypertension	1.89 (1.08–3.30)	0.025
Laparotomy	1.64 (1.03–2.59)	0.036
Small bowel enterotomy	3.02 (1.26–7.21)	0.013
Lysis of bowel adhesions	1.63 (0.93–2.86)	0.090
Hysterectomy	2.59 (1.62–4.15)	< 0.001
Ureterolysis	1.49 (0.68–3.28)	0.322

**Table 4:** Comparison of women with and without major complications following laparoscopic colorectal resection for endometriosis

**Characteristics**	**No major complications (*n*** **= 446)**	**Major complications (*n*** **= 49)**	***p*-value**
Age (years)	40.0 [35.0–46.0]	40.0 [36.0–43.0]	0.326
Race			0.039
White	293 (65.7)	21 (42.9)	
Black or African American	56 (12.6)	12 (24.5)	
Hispanic	28 (6.3)	7 (14.3)	
Asian	12 (2.7)	3 (6.1)	
Native Hawaiian or Pacific Islander	1 (0.2)	0 (0)	
American Indian or Alaska Native	3 (0.7)	0 (0)	
Unknown	53 (11.9)	6 (12.2)	
Body mass index, mean (kg/m^2^)	26.2 [22.9–31.6]	26.6 [22.6–32.4]	0.906
Tobacco use	36 (8.1)	6 (12.2)	0.320
Diabetes mellitus	9 (2.0)	1 (2.0)	< 0.999
Hypertension	61 (13.7)	12 (24.5)	0.043
Chronic obstructive pulmonary disease	2 (0.4)	1 (2.0)	0.173
Immunosuppressive therapy	10 (2.2)	2 (4.1)	0.337
Bleeding disorders	3 (0.7)	0 (0)	> 0.999
Preoperative transfusion	0 (0)	1 (2.0)	0.099
ASA classification			0.804
I	65 (14.6)	5 (10.2)	
II	310 (69.5)	37 (75.5)	
III	70 (15.7)	7 (14.3)	
Unknown	1 (0.2)	0 (0)	
Colectomy	439 (98.4)	48 (98.0)	0.568
Rectal resection	7 (1.6)	1 (2.0)	
Colostomy	1 (0.2)	0 (0)	> 0.999
Concomitant procedures			
Small bowel enterotomy	8 (1.8)	3 (6.1)	0.085
Lysis of bowel adhesions	49 (11.0)	15 (30.6)	< 0.001
Other bowel procedures	4 (0.9)	0 (0.0)	> 0.999
Hysterectomy	77 (17.3)	16 (32.7)	0.009
Myomectomy	12 (2.7)	4 (8.2)	0.063
Ovarian cystectomy	10 (2.2)	4 (8.2)	0.04
Salpingectomy w/wo oophorectomy	90 (20.2)	18 (36.7)	0.008
Excision or fulguration of pelvic lesions	111 (24.9)	19 (38.8)	0.036
Ureterolysis	16 (3.6)	6 (6.1)	0.381
Total operative time (minutes)	197.0 [126.8–296.3]	303.0 [233.5–442.0]	< 0.001
Minor complication	12 (2.7)	6 (12.2)	0.001

Data are *n* (%) or median [interquartile range]

In subanalysis multivariable regression of laparoscopic surgeries only (Table 5), Hispanic ethnicity (aOR 95%CI 3.78 (1.44–9.96), *p* = 0.007), chronic hypertension (aOR 95%CI 2.35 (1.06–5.20), *p* = 0.036), lysis of bowel adhesions (aOR 95%CI 2.96 (1.40–6.28), *p* = 0.005), and hysterectomy (aOR 95%CI 2.13 (1.08–4.21), *p* = 0.029) were independently positively associated with major complications.

**Table 5:** Multivariable regression analysis of factors associated with major complications following laparoscopic colorectal resection for endometriosis

	**Odds ratio 95% confidence interval**	***p*-value**
Race and ethnicity		
White	Reference	**–**
Black or African American	2.12 (0.94–4.80)	0.070
Hispanic	3.78 (1.44–9.96)	0.007
Asian	3.02 (0.73–12.47)	0.127
Hypertension	2.35 (1.06–5.20)	0.036
Lysis of bowel adhesions	2.96 (1.40–6.28)	0.005
Hysterectomy	2.13 (1.08–4.21)	0.029
Laparoscopic excision or fulguration of pelvic lesions	1.60 (0.79–3.21)	0.189

In subanalysis multivariable regression of laparotomies only (Table S2), Black race (aOR 95%CI 4.31 (1.95–9.52), *p* < 0.001) and hysterectomy (aOR 95%CI 3.38 (1.72–6.63), *p* < 0.001) were independently positively associated with major complications.

## Discussion

The overall rate of major complications following colorectal resections for endometriosis was 13.5%. The rate of major complications after laparotomy was 20.4% and after laparoscopy 9.9%. Black race, Hispanic ethnicity, chronic hypertension, laparotomy, concomitant small bowel enterotomy or lysis of bowel adhesions, and hysterectomy were associated with increased risk of major complications following colorectal resections for endometriosis.

The rate of major complications in prior reports varies widely, and comparison to our results is hindered by variation in study designs, complications definitions, and follow-up periods. A systematic review of 34 studies reported an overall major complication rate of 11% including severe bowel complications, hemorrhage, and infections [12]. In another review of 36 studies on surgical treatment of rectovaginal endometriosis, the rate of major complications was 3–10% [14]. Reported complications were both short- and long-term. A recent systematic review and meta-analysis reported an overall complication rate of 9.9% after colorectal segmental resection. However, the definition of complications differed between studies and included mostly long-term outcomes [6]. In a NSQIP study of 12,455 laparoscopic and 33,190 open colectomy cases, the rates of Clavien-Dindo 4 and 5 complications were 3.6% and 15.4% respectively [15, 16]. Of note, 35% of that study population represented cases with malignancy. A prior NSQIP study on colorectal resection for endometriosis found a 7.1% major complications rate [13]. However, in that study, data were collected between 2005 and 2014, and blood transfusion was not considered a major complication, which occurred in 55.9% of our major complications group. Thus, when excluding blood transfusions, the overall complication rate in our study is in line with that previous report, suggesting a stable rate of complications during the last two decades.

Overall, laparoscopic surgery was performed in 65.6% of cases, at a gradually increasing rate between 2012 and 2020. The average rate is lower than the 82.3% rate reported in another NSQIP study examining the years 2014 to 2019 [17] but is higher than the rate reported in a NSQIP study on colorectal surgeries for endometriosis from 2005 to 2014 (53.7%) [13]. This trend may represent a shift favoring minimally invasive surgery throughout the years. A systematic review of 60 studies on colorectal surgeries for endometriosis found that more than 98% of cases were performed laparoscopically [6]. Possibly, centers reporting outcomes following colorectal resections for endometriosis have a higher level of expertise in treating endometriosis laparoscopically. The NSQIP database, in contrast, may better represent an average expertise in colorectal endometriosis treatment.

We performed separate sub-analyses for open and laparoscopic surgeries, as laparotomy is an established risk factor for surgical complications compared to a minimally invasive approach, and adjusting for this route of surgery in a multivariable regression may have some limitations [18, 19]. Indeed, we found a 1.64 aOR for the risk of major complications following laparotomy when evaluating the entire cohort. Furthermore, when evaluated separately, independent risk factors for complications differed between the open and minimally invasive groups. Interestingly, a prior NSQIP study did not find a different complication rate when comparing the two surgical approaches [13]. Possibly, statistical power was limited by the sample size (*n* = 268).

Any bowel procedure may increase the risk of complications and should be taken into account when studying surgical outcomes [6, 20, 21]. We found that concomitant small bowel enterotomy was associated with increased complications risk in the general analysis. This finding is not surprising, as any additional enterotomy can increase the risk of complications. Of note, the NSQIP database does not allow for separation between planned and unplanned enterotomies. This fact should be taken into consideration, as the possibility of unplanned contaminated surgery due to small bowel injury, and subsequent increased risk of infection, cannot be ruled out. Lysis of bowel adhesions was associated with complications in the laparoscopic sub-group analysis, while small bowel enterotomy was not, probably due to the small sample size. Bowel adhesiolysis, including mobilization, has been associated with increased surgical complexity and morbidity, in line with our results [22].

Concomitant hysterectomy at the time of colorectal resection was independently associated with major complications in the three multivariable analyses. Prior studies reported higher complication risk in surgeries where a hysterectomy was performed compared with uterine preserving surgeries [23–25] In addition, cases where a hysterectomy was performed may represent more advanced disease and/or reoperation following prior intervention, which may increase the risk of complications [1].

We found that Black race and Hispanic ethnicity, compared to White race, were associated with increased risk for major complications in the general analysis. Our results are in line with previous studies. A recent NSQIP-based study on women undergoing surgery for endometriosis found an increased complication rate among Hispanic, Black, Pacific Islanders, and Native Americans compared to White women [26]. Another NSQIP study on hysterectomies for endometriosis found that the Black race was independently associated with major complications compared to the White race, as was found in the subanalysis of laparotomies only [17]. Race disparity has also been reported in gynecologic surgeries performed for different indications. A NSQIP-based study found an increased risk of morbidity following myomectomy among Black women compared with White women [27]. Another study based on a state database found an increased risk of complications after myomectomy among women of Black and Asian race [28]. In the subanalysis of laparoscopic surgeries, the Black race was no longer associated with increased complications risk, while the Hispanic race remained associated. Possibly, the adjustment for laparotomies and hypertension, which occurred in high proportions among Black patients in our cohort, accounts for the difference between the univariate and multivariable analyses. Among Hispanic patients, the proportion of hypertension was similar in both groups, possibly accounting for the fact that ethnicity remained significantly associated with complications after multivariable regression analysis. Interestingly, Hispanic ethnicity was no longer an independent risk factor for complications in the subanalysis of laparotomies only, most probably due to a small sample size of this group.

Limited access to surgeon experts in treating colorectal endometriosis may be one of the underlying causes of the disparity in outcomes [29, 30]. Further research focusing on preoperative evaluation, surgical decision-making, and surgical practice in colorectal endometriosis surgeries for endometriosis among patients from different races and ethnicities is warranted. Our results may portray a reliable state of the proportion of open and minimally invasive surgeries for colorectal endometriosis, in contrast to proportions reported in publications from high-volume expert medical centers. Thus, evaluation of the surgical approach in the treatment of deep infiltrating endometriosis should also be evaluated in an effort to reduce the proportion of laparotomies.

Our study has several important limitations. Outcomes reported in the NSQIP database are limited to 30 days post-operation. Significant long-term outcomes are thus not included in this study. In addition, important parameters, which may have affected the results, are not reported in the NSQIP database (e.g., prior surgeries, preoperative imaging, surgeons’ surgical volume, intraoperative findings). Moreover, we could not specify whether some of the included procedures, for example, concomitant small bowel resection, were planned or unplanned, limiting the conclusions that can be drawn from the results. In addition, the CPT code 58,662 (laparoscopy, surgical; with fulguration or excision of lesions of the ovary, pelvic viscera, or peritoneal surface by any method) may be used by clinicians to describe bowel surgery. Thus, the proportion of concomitant intestinal or rectal procedures in our results may be an underestimation. In addition, data on conversion from the laparoscopic approach to laparotomy are not provided in the NSQIP database, limiting the possibility of studying the risk factors for this complication. Furthermore, we could not sub-categorize robotic-assisted and laparoscopic surgeries, potentially introducing bias.

The strengths of this study include the use of a large, validated surgical database, allowing the examination of specific surgical outcomes. More than 700 hospitals participated in the American College of Surgeons NSQIP project in 2020. Thus, although this dataset does not represent a nationally representative sample, the results may be generalizable to women undergoing surgery in the USA. Given that laparotomy is a major risk factor for post-operative complications, the separate sub-analyses of laparoscopic and laparotomies are another strength of this study.

## Conclusion

This study provides a current population-based estimate of short-term complications after laparoscopy or laparotomy for colorectal endometriosis in the USA. The identified risk factors for complications can assist during preoperative shared decision-making and informed consent process. This study can serve as a reference for further studies on racial and ethnic disparity and outcomes, which should focus on the association between race and ethnicity and complications following colorectal surgery for endometriosis.

## Supplementary Information

Below is the link to the electronic supplementary material.

Supplementary file1 (DOCX 22 KB)

## Data Availability

The datasets generated during and/or analyzed during the current study are available from the corresponding author on reasonable request.
