# Supplementary material for: Risk factors for major complications following colorectal resections for endometriosis in the USA
Source: Int J Colorectal Dis. 2023 Dec 6;39(1):1. doi: 10.1007/s00384-023-04577-5 (PMC10700479; doi:10.1007/s00384-023-04577-5)
Supplement: Supplementary file 1 — Supplementary file1 (DOCX 22 KB) [file 384_2023_4577_MOESM1_ESM.docx]

**Table S1**. Comparison of women with and without major complications following laparotomy for colorectal resection for endometriosis

| **Characteristics** | **No major complications (n=207)** | **Major complications (n=53)** | **p value** |
| --- | --- | --- | --- |
| Age, years | 41.0 [34.0-45.0] | 41.0 [35.5-45.5] | 0.473 |
| Race  White  Black or African American  Hispanic  Asian  Native Hawaiian or Pacific Islander  Unknown | 126 (60.9)  32 (15.5)  13 (6.3)  12 (5.8)  2 (1.0)  22 (10.6) | 19 (35.8)  19 (35.8)  5 (9.4)  3 (5.7)  1 (1.9)  6 (11.3) | **0.012** |
| Body mass index, mean, kg/m2 | 26.4 [22.6-31.0] | 25.6 [23.3-35.5] | 0.425 |
| Tobacco use | 20 (9.7) | 6 (11.3) | 0.719 |
| Diabetes mellitus | 3 (1.4) | 4 (7.5) | **0.034** |
| Hypertension | 29 (14.0) | 12 (22.6) | 0.124 |
| Chronic obstructive pulmonary disease | 0 (0) | 0 (0) | - |
| Immunosuppressive therapy | 3 (1.4) | 3 (2.8) | 0.270 |
| Bleeding disorders | 2 (1.0) | 0 (0) | >0.999 |
| Preoperative transfusion | 1 (0.5) | 1 (1.9) | 0.367 |
| ASA classification  I  II  III  IV | 24 (11.6)  147 (71.0)  35 (16.9)  1 (0.5) | 6 (11.3)  32 (60.4)  15 (28.3)  0 (0) | 0.284 |
| Colectomy  Rectal resection | 176 (85.0)  31 (15.0) | 44 (83.0)  9 (17.0) | 0.718 |
| Colostomy | 5 (2.4) | 3 (5.7) | 0.209 |
| Concomitant procedures   - Small bowel enterotomy - Lysis of bowel adhesions - Other bowel procedures - Hysterectomy - Myomectomy - Ovarian cystectomy - Salpingectomy w/wo oophorectomy - Excision or fulguration of pelvic lesions - Ureterolysis | 11 (5.3)  37 (17.9)  9 (4.3)  66 (31.9)  5 (2.4)  9 (4.3)  93 (44.9)  26 (12.6)  14 (6.8) | 9 (17.0)  11 (20.8)  4 (7.5)  31 (58.5)  1 (1.9)  0 (0)  38 (71.7)  4 (7.5)  8 (15.1) | **0.004**  0.630  0.309  **<0.001**  >0.999  0.211  **0.001**  0.308  0.052 |
| Total operative time, minutes | 214.0 [159.0-282.0] | 279.0 [213.0-370.0] | **<0.001** |
| Minor complication | 17 (8.2) | 6 (11.3) | 0.477 |

Data are n (%) or median [interquartile range]

**Table S2**. Multivariable regression analysis of factors associated with major complications following laparotomy for colorectal resection for endometriosis.

|  | **Odds Ratio 95% Confidence interval** | **p value** |
| --- | --- | --- |
| Race and ethnicity |  |  |
| White | Reference | **-** |
| Black or African American | 4.31 (1.95-9.52) | **<0.001** |
| Hispanic | 2.05 (0.63-6.66) | 0.233 |
| Asian | 1.46 (0.36-5.88) | 0.595 |
| Diabetes mellitus | 3.90 (0.66-22.88) | 0.132 |
| Hysterectomy | 3.38 (1.72-6.63) | **<0.001** |

**Table S3.** Current procedural terminology codes of concomitant intestinal and rectal procedures.

| **Intestinal Surgery** 44005-44055: Under Incision Procedures on the Intestines (Except Rectum)  44100-44139: Under Excision Procedures on the Intestines (Except Rectum) 44602-44680: Under Repair Procedures on the Intestines (Except Rectum) 44180: Under Laparoscopic Incision Procedures on the Intestines (Except Rectum) 44227: Under Laparoscopic Repair Procedure on the Intestines (Except Rectum) |
| --- |
| **Rectal Surgery** 45000-45020: Under Incision Procedures on the Rectum 45100-45109: Under Excision Procedures on the Rectum 45500-45825: Under Repair Procedures on the Rectum 45398-45399: Under Excisional Laparoscopic Procedures on the Rectum 45400-45499: Under Laparoscopic Repair Procedures on the Rectum |
